# One-Dimensional Hydrogen-Bonded Infinite Chain from Nickel(II) Tetraaza Macrocyclic Complex and 1,2-Cyclopentanedicarboxylate Ligand

**DOI:** 10.3390/ijms12042232

**Published:** 2011-04-01

**Authors:** In-Taek Lim, Ki-Young Choi

**Affiliations:** Department of Chemistry Education, Kongju National University, Kongju 314-701, Korea; E-Mail: hak124@kongju.ac.kr

**Keywords:** 1D hydrogen-bonded infinite chain, nickel(II) complex, tetraaza macrocycle, *trans*-1,2-cyclopentanedicarboxylic acid, distorted octahedral geometry

## Abstract

The reaction of [Ni(L)]Cl_2_·2H_2_O (L = 3,14-dimethyl-2,6,13,17-tetraazatricyclo [14,4,0^1.18^,0^7.12^]docosane) with *trans*-1,2-cyclopentanedicarboxylic acid (H_2_-cpdc) yields a 1D hydrogen-bonded infinite chain with formula [Ni(L)(H-cpdc^−^)_2_] (**1**). This complex has been characterized by X-ray crystallography, spectroscopy and cyclic voltammetry. The crystal structure of **1** exhibits a distorted octahedral geometry about Ni atom with four nitrogen atoms of the macrocycle and two oxygen atoms of the H-cpdc^−^ ligand at the axial position. Compound **1** crystallizes in the monoclinic system *P*2_1_/c with *a* = 8.7429(17), *b* = 10.488(2), *c* = 18.929(4) Å, β = 91.82(2), *V* = 1734.8(6) Å^3^, *Z* = 2. Electronic spectrum of **1** reveals a high-spin octahedral environment. Cyclic voltammetry of **1** undergoes two waves of a one-electron transfer corresponding to Ni^II^/Ni^III^ and Ni^II^/Ni^I^ processes.

## 1. Introduction

The multidimensional supramolecules self-assembled by metal ions and mutidentate organic ligands have been of great interest due to specific structural features and potential applications such as catalysts, electronic conductivities, optical properties, and molecular magnets [1–9]. In the self-assembly of the supramolecular networks, intermolecular forces such as hydrogen bonds and π-π interactions are usually involved together with metal-ligand coordination bonds [10–13]. Especially, hydrogen bonding is one of the key interactions for the process of molecular aggregation and recognition in nature, which creates novel structures of molecular assemblies [14]. In particular, self-assembly of macrocyclic complexes containing the square-planar geometry with aromatic and aliphatic polycarboxylate ligands has been proved to be good building blocks for the construction of coordination polymers and metallosupramolecules [15–20]. For example, 1D nickel(II) complexes {[Ni(L)(tp)]·2H_2_O}*_n_* (*L* = 3,14-dimethyl-2,6,13,17-tetraazatricyclo[14,4,0^1.18^,0^7.12^]docosane; *tp* = terephthalate) [15] and {[Ni(L)(isotp)]·3H_2_O}*_n_* (isotp = isophthalate) [16] show distorted octahedral geometries and reveal weak antiferromagnetic interactions, which are assembled by square-planar nickel(II) complex [Ni(L)]Cl_2_·2H_2_O and aromatic dicarboxylate ligands. Furthermore, the reaction of [Ni(L)]Cl_2_·2H_2_O with 1,3,5-benzenetricarboxylic acid (H_3_-btc) yields 2D nickel(II) complex via covalent and hydrogen bonds {[Ni(L)]_3_[μ-btc]_2_·8H_2_O}*_n_* [15], which reveals a geometrically symmetric core with a {4/6} coordination number set. In addition, the octahedral nickel(II) complexes {[Ni(L)(oxalato)]·H_2_O}*_n_* [17] and [Ni(L)(malonato)]*_n_* [18] show that the nickel(II) ions in the complexes are bridged by the aliphatic dicarboxylate ligands to form 1D coordination polymers. However, the cyclo-aliphatic nickel(II) complexes [Ni(hatt)(H-chdc^–^)_2_] (hatt = 1,3,10,12,16,19-hexaazatetracyclo[17,3,1,1^12.16^,0^4.9^]tetracosane; H_2_-chdc = *trans*-1,2-cyclohexanedicarboxylic acid) [19] and [Ni(L)(H-cbdc^–^)_2_] (H_2_-cbdc = 1,1-cyclobutanedicarboxylic acid) [20] show the distorted octahedral geometries, which assemble in the solid state to form 1D hydrogen polymers. Therefore, the hydrogen-bonding interactions play a significant role in aligning the molecules and polymer stands in the crystalline solids.

To further investigate the coordination behavior, we attempted to self-assemble [Ni(L)]Cl_2_·2H_2_O containing the *trans*-1,2-cyclopentanedicarboxylic acid as building block. Herein, we report and characterize a 1D hydrogen-bonded infinite chain [Ni(L)(H-cpdc^–^)_2_] (**1**) (L = 3,14-dimethyl-2,6,13,17-tetraazatricyclo[14,4,0^1.18^,0^7.12^]docosane; H_2_-cpdc = *trans*-1,2-cyclopentanedicarboxylic acid).

**Figure:**
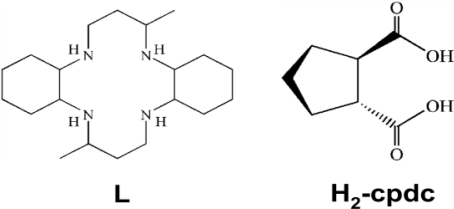


## 2. Results and Discussion

### 2.1. Structural Description

**Figure 1.**
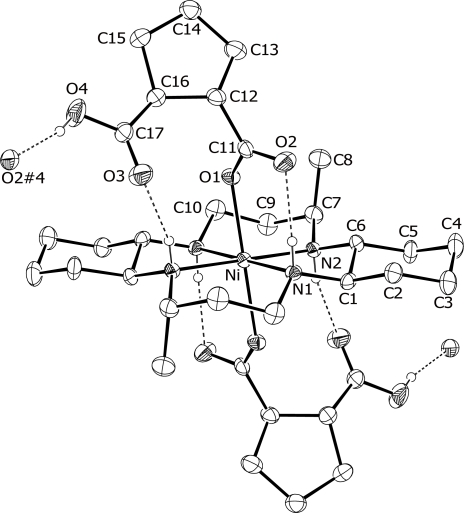
An ORTEP diagram of [Ni(L)(H-cpdc**^−^**)_2_] (**1**) with the atomic numbering scheme (30% probability ellipsoids shown). Symmetry code: (#4) −x, y − 1/2, −z **+** 3/2.

An ORTEP drawing [21] of [Ni(L)(H-cpdc^–^)_2_] (**1**) with the atomic numbering scheme is shown in Figure 1. Selected bond distances and angles are listed in Table 1. The skeleton of the macrocyclic unit in **1** adopts the classical *trans-*III (*R*,*R*,*S*,*S*) conformation with two chair-form six-membered and two gauche-form five-membered chelate rings. The central nickel atom is located on an inversion center. The nickel atom and the four nitrogen atoms of the macrocycle are exactly in a plane. The nickel(II) ion exhibits a distorted octahedral coordination geometry with the four secondary amine nitrogen atoms of the macrocycle and two oxygen atoms of H-cpdc^–^ ligands in axial positions. The average Ni-N distance of 2.063(4) Å is significantly longer than in the square-planar geometry of [Ni(L)]Cl_2_·2H_2_O [1.948(4) Å] [22], but is similar to those observed for high-spin octahedral nickel (II) complexes with 14-membered macrocyclic ligands [15–20]. The Ni-O distance of 2.176(4) Å is similar to those previously reported values in closely related examples {[Ni(L)]_3_[μ-btc]_2_·8H_2_O}*_n_* (btc^3−^ = 1,3,5-benzenetricarboxylate, 2.193(4) and 2.163(4) Å) [15], {[Ni(L)(H-chtc)]·H_2_O}*_n_* (chtc^3−^ = 1,3,5-cyclohexanetricarboxylate, 2.176(6) and 2.152(6) Å) [16], in which these complexes are 2D coordination and hydrogen-bonded infinite chains. The N-Ni-N angles of the six-membered chelate rings are larger than those of the five-membered chelate rings. The dihedral angle between the plane of the carboxylate group and NiN_4_ plane is 67.6(5)°. The closest intermolecular Ni···Ni distance between neighboring stands is 8.743(2) Å. The Ni-O(1) linkage is bent slightly off the perpendicular to the NiN_4_ plane by 3.2–7.0°. The Ni-O(1)-C(11) and O(1)-C(11)-O(2) angles related to the H-cpdc^−^ ligand are 130.1(4) and 122.9(6)°, respectively. The deprotonated one among the two H-cpdc^–^ carboxylic groups is coordinated to the metal center. The secondary amines N(1) and N(2) of the macrocycle are intramolecular hydrogen bonded to the uncoordinated carboxylic oxygen O(2) and O(3) of the H-cpdc^–^ ligand [N(1)-H(17)^…^O(2)#3 2.797(6) Å, 163(6)°; N(2)-H(18)^…^O(3)#2 3.085(7) Å, 158(6)°; symmetry codes (#2) −x, −y + 1, −x + 1; (#3) x, y, z].

**Figure 2.**
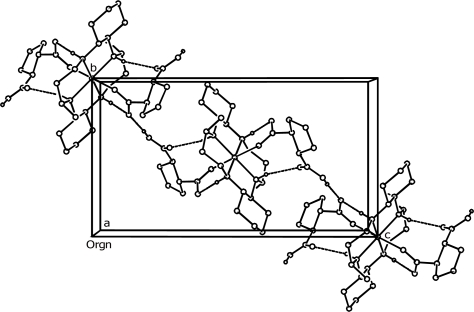
Crystal packing diagram of [Ni(L)(H-cpdc^–^)_2_] (**1**), showing the intermolecular hydrogen bonds as dashed lines The hydrogen atoms other than those participating in hydrogen bonding are omitted for clarity.

Interestingly, the protonated oxygen atom O(4) of the H-cpdc^–^ ligand forms intermolecular hydrogen bond to an adjacent uncoordinated carboxylic oxygen O(2) of the H-cpdc^–^ ligand [O(4)-H(19)^…^O(2)#4 2.522(7) Å, 166(10)°; symmetry code (#4) −x, y − 1/2, −z + 3/2] (Figure 2 and Table 2). This interaction gives rise to a 1D hydrogen-bonded infinite chain, which is similar to that observed for the cyclo-aliphatic nickel(II) complexes [Ni(hatt)(H-chdc^–^)_2_] [19] and [Ni(L)(H-cbdc^–^)_2_] [20]. This fact may be due to the flexibility of the *trans*-1,2-cyclopentanedicarboxylate (H-cpdc^–^) ligand as well as *trans*-1,2-cyclohexanedicarboxylate (H-chdc^–^) and1,1-cyclobutanedicarboxylate (H-cbdc^–^) ligands.

### 2.2. Chemical Properties

**Figure 3.**
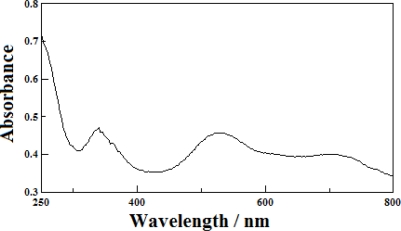
Solid state electronic absorption spectrum of [Ni(L)(H-cpdc^–^)_2_] (**1**) by the diffuse reflectance method at 20.0 ± 0.1 °C.

The IR spectrum of **1** shows a band at 3140 cm^−1^ corresponding to the ν(NH) of the coordinated secondary amines of the macrocycle. Two strong bands exhibit ν_as_(COO) stretching frequency at 1561 cm^−1^ and ν_sym_(COO) at 1396 cm^−1^, respectively. The value of Δν (165 cm^−1^) indicates that the carboxylate groups coordinated to the nickel(II) ion only as a monodentate ligand [23,24]. In addition, a sharp band at 3426 cm^−1^ is associated to the ν(OH) stretching vibration of the hydroxyl group in the H-cpdc^–^ ligand. The UV-Vis spectrum of **1** is listed in Table 3. The UV spectrum of **1** in the water solution shows an absorption maximum in the region 260 nm attributed to a ligand-metal charge transfer associated with the nitrogen and oxygen donors [25]. As shown in Figure 3, the solid state electronic spectrum of **1** in the visible region shows three absorption bands at 340, 530, and 694 nm assignable to the ^3^B_1g_ → ^3^E_g_^c^, ^3^B_1g_ → ^3^E_g_^b^, ^3^B_1g_ → ^3^B_2g_ + ^3^B_1g_ → ^3^A_2g_^a^ transitions, which is the characteristic spectrum expected for a high-spin d^8^ nickel(II) ion in a distorted octahedral environment [26,27]. However, the complex **1** dissolves in water and decomposes into the original compound [Ni(L)](ClO_4_)_2_ (459 nm) [28], which has a low-spin d^8^ nickel(II) ion in a square-planar environment. This fact can be understood in terms of the decomposition of the building block in water solution. The electronic spectrum for **1** clearly supports the structure determined by the X-ray diffraction study.

**Figure 4.**
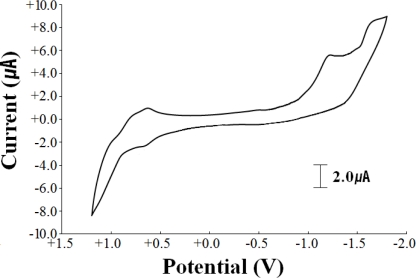
Cyclic voltammogram of [Ni(L)(H-cpdc^−^)_2_] (**1**) in 0.1 M TEAP-DMSO solution at 20.0 ± 0.1 °C. The scan rate is 100 mV/s.

Cyclic voltammetric data for **1** in 0.10 M TEAP-DMSO solution are given in Table 4. Cyclic voltammogram of **1** in 0.1 M TEAP-DMSO solution is shown in Figure 4. The oxidation and reduction potentials for **1** give the irreversible and reversible one-electron processes at +0.66 and −1.23 V *versus* the Ag/AgCl reference electrode, assigned to the Ni^II^/Ni^III^ and Ni^II^/Ni^I^ couples, respectively. This fact may be attributed to the coordination of the axial H-cpdc^−^ ligand, which is in agreement with the crystal structure of **1**.

## 3. Experimental Section

### 3.1. Materials and Methods

All chemicals and solvents used in the syntheses were of reagent grade and were used without further purification. The complex [Ni(L)]Cl_2_·2H_2_O was prepared according to literature method [22]. IR spectra were recorded with a Perkin-Elmer Paragon 1000 FT-IR spectrophotometer using KBr pellets. Solution and solid electronic spectra were obtained on a JASCO Uvidec 610 spectrophotometer. Electrochemical measurements were accomplished with a three electrode potentiostat BAS-100BW system. A 3 mm Pt disk was used as the working electrode. The counter electrode was a coiled Pt wire and a Ag/AgCl electrode was used as a reference electrode. Cyclic voltammetric data were obtained in DMSO solution with 0.10 M tetraethylammonium perchlorate (TEAP) as supporting electrolyte at 20.0 ±0.1 °C. The solution was degassed with high purity N_2_ prior to carrying out the electrochemical measurements. Elemental analyses (C, H, N) were performed on a Perkin-Elmer CHN-2400 analyzer.

### 3.2. Synthesis of [Ni(L)(H-cpdc^–^)_2_] (**1**)

To a methanol solution (20 cm^3^) of [Ni(L)]Cl_2_·2H_2_O (251 mg, 0.5 mmol) sodium *trans*-1,4-cyclohexanedicarboxylate was added (108 mg, 0.5 mmol) and the mixture was stirred for 30 min at room temperature. The solution was filtered to remove insoluble material. After the filtrate was allowed to stand at room temperature over a period of several days, violet crystals formed Crystals were collected by filtration and washed with diethyl ether. Anal. Calcd. for C_34_H_58_N_4_NiO_8_: C, 57.55; H, 8.24; N, 7.90. Found: C, 57.64; H, 8.32; N, 7.81%. IR (KBr, cm^−1^): 3426(m), 3191(m), 3140(m), 2931(m), 2861(s), 1630(m), 1561(s), 1448(m), 1396(s), 1308(m), 1268(w), 1155(w), 1111(s), 1076(m), 997(m), 948(m), 897(m), 786(w), 719(w), 639(w), 552(w), 537(w).

### 3.3. X-ray Crystallography

Single crystal X-ray diffraction measurement for **1** was carried out on an Enraf-Nonius CAD4 diffractometer using graphite-monochromated Mo-Kα radiation (λ = 0.71073 Å). Intensity data were measured at 293(2) K by ω-2θ technique. Accurate cell parameters and an orientation matrix were determined by the least-squares fit of 25 reflections. The intensity data were corrected for Lorentz and polarization effects. Empirical absorption correction was carried out using φ-scan [29]. The structure was solved by direct methods [30] and the least-squares refinement of the structure was performed by the SHELXL-97 program [31]. All non-hydrogen atoms were refined anisotropically. The hydrogen atoms were placed in calculated positions allowing them to ride on their parent C atoms with *U*_iso_(H) = 1.2*U*_eq_(C or N). Crystal parameters and details of the data collections and refinement are summarized in Table 4.

## 4. Conclusions

The reaction of [Ni(L)]Cl_2_·2H_2_O (L = 3,14-dimethyl-2,6,13,17-tetraazatricyclo[14,4,0^1.18^,0^7.12^]docosane) with *trans*-1,2-cyclopentanedicarboxylic acid (H_2_-cpdc) yields a 1D hydrogen-bonded infinite chain, which exhibits a distorted octahedral geometry with four nitrogen atoms of the macrocycle and two oxygen atoms of the *trans*-1,2-cyclopentanedicarboxylate ligand at the axial position. The hydrogen-bonding interactions of **1** play a significant role in aligning the polymer stands. Solid state electronic absorption spectrum of **1** reveals a high-spin d^8^ nickel(II) ion in a distorted octahedral environment. Cyclic voltammetry of **1** undergoes two waves of a one-electron transfer corresponding to Ni^II^/Ni^III^ and Ni^II^/Ni^I^ processes. This complex makes the oxidation of Ni(II) to Ni(III) easier and the reduction to Ni(I) more difficult. This fact may be attributed to the coordination of the axial H-cpdc^−^ ligand, which is in agreement with the crystal structure of **1**.

## Figures and Tables

**Table 1.** Selected bond distances (Å) and angles (°) for [Ni(L)(H-cpdc^–^)_2_] (**1**).

***Bond Lengths***			
Ni-N(1)	2.043(5)	Ni-N(2)	2.082(5)
Ni-O(1)	2.176(4)	O(1)-C(11)	1.259(7)
O(2)-C(11)	1.253(7)	O(3)-C(17)	1.203(7)
O(4)-C(17)	1.290(8)	Ni^…^Ni#1	8.743(2)
***Bond Angles***			
N(1)-Ni-N(2)	83.8(2)	N(1)-Ni-N(2)#2	96.2(2)
N(1)-Ni-O(1)	93.2(2)	N(1)#2-Ni-O(1)	86.8(2)
N(2)-Ni-O(1)	97.0(2)	N(2)#2-Ni-O(1)	83.0(2)
Ni-O(1)-C(11)	130.1(4)	O(1)-C(11)-O(2)	122.9(6)
O(3)-C(4)-O(4)	122.0(6)		

Symmetry codes: (#1) x + 1, y, z; (#2) −x, −y + 1, −x + 1.

**Table 2.** Hydrogen bonding parameters (Å, °) for [Ni(L)(H-cpdc^–^)_2_] (**1**).

**D-H^…^A**	**D-H (Å)**	**H^…^A (Å)**	**D^…^A (Å)**	**∠D-H^…^A (°)**
N(1)-H(17)^…^O(2)#3	0.85(6)	1.97(6)	2.797(6)	163(6)
N(2)-H(18)^…^O(3)#2	0.82(6)	2.31(6)	3.085(7)	158(6)
O(4)-H(19)^…^O(2)#4	0.79(9)	1.75(9)	2.522(7)	166(10)

Symmetry codes: (#2) −x, −y + 1, −x + 1; (#3) x, y, z; (#4) −x, y − 1/2, −z + 3/2.

**Table 3.** Electronic spectral data ^a^.

**Complex**	**State**	**λ_max_/nm (**ɛ**/M^−1^ cm^−1^)**
[Ni(L)](ClO_4_)_2_·2H_2_O ^b^	MeCN	465(66)
	H_2_O	459(70)
[Ni(L)(H-cpdc**^−^**)_2_] (**1**)	Solid	340, 530, 694
	H_2_O	260(2.5 × 10^2^), 458(67)

a Solution = H_2_O at 20 ±0.1 °C; Solid = diffuse reflectance;

b Reference [28].

**Table 4.** Cyclic voltammetric data ^a^.

	**Potentials (V) *versus* Ag/AgCl**	
Complex	Ni(II)/Ni(III)	Ni(II)/Ni(I)
[Ni(L)](ClO_4_)_2_^b^	+0.73	−1.63
[Ni(L)(H-cpdc^−^)_2_] (**1**)	+0.66 (i) ^c^	−1.23

a Measured in 0.10 M TEAP-DMSO solution at 20.0 ± 0.1 °C;

b Reference [28]. These values are reduced from those of Ag/AgCl reference electrodes;

c *i* = irreversible.

**Table 4.** Crystallographic data for [Ni(L)(H-cpdc^–^)_2_] (**1**).

**Empirical Formula**	**C_34_H_58_N_4_NiO_8_**
Formula weight	709.55
Temperature (K)	293(2)
Crystal system	Monoclinic
Space group	*P*2_1_/c
*a* (Å)	8.7429(17)
*b* (Å)	10.488(2)
*c* (Å)	18.929(4)
β (°)	91.82(2)
*V* (Å^3^)	1734.8(6)
*Z*	2
*D*_calc_ (Mg m^−3^)	1.358
Absorption coefficient (mm^−1^)	0.615
*F*(000)	764
Crystal size (mm)	0.30 × 0.20 × 0.10
θ range (°)	2.15 to 24.99
Limiting indices	−10 ≤ *h* ≤ 10, −1 ≤ *k* ≤ 12, −1 ≤ *l* ≤ 22
Reflection collected	3476
Reflection unique	3055
Absorption correction	φ-scan
Max./min. transmission	0.9403 and 0.8192
Parameters	223
Goodness of fit on *F*^2^	1.136
Final *R* indices [*I* > 2σ(*I*)]	*R*_1_^a^ = 0.0628, *wR*_2_^b^ = 0.1617
*R* indices (all data)	*R*_1_ = 0.1573, *wR*_2_ = 0.1855
Largest difference peak and hole (eÅ^−3^)	0.366 and −0.338

a *R*_1_ = Σ||*F*_o_|–|*F*_c_||/Σ|*F*_o_|;

b *R*_2_ = [Σ[*w*(*F*_o_^2^ − *F*_c_^2^)^2^]/Σ[*w*(*F*_o_^2^)^2^]]^1/2^.

## Supplementary Material

Crystallographic data for the structural analysis have been deposited with the Cambridge Crystallographic Data Center, CCDC No. 812214 for **1**. Copies of this information may be obtained free of charge from the Director, CCDC, 12 Union Road, Cambridge, CB2, 1EZ, UK (Fax: +44-1223-336033; E-Mail: deposit@ccdc.cam.uk or http://www.ccdc.cam.ac.uk).
